# Died in a Dentist's Chair

**Published:** 1896-01

**Authors:** 


ARTICLE VI.

### DIED IN A DENTIST'S CHAIR.

Mrs. Charles Grier died yesterday afternoon shortly after three o'clock while in a dentist's chair in the office of Billings & Sherraden, dentists. She went to have teeth extracted, and was put under the influence of chloroform. It was from the effects of chloroform that she died.

Mrs. Grier went to the office shortly before 1 o'clock, and was attended by Dr. Sherraden. She had two teeth pulled and then said the operation pained her too much, and said she wished to be placed under the influence of an anæsthetic. Dr. Sherraden told her that he would not place her under the influence of any drug, and tried to induce her to continue the operation. Dr. Arnold, who has an office in the building, was called and examined Mrs. Grier. He told her that she could safely take chloroform, but refused to administer it. Then Mrs. Grier asked to have Dr. R. C. Moore, who had attended her in the past summoned.

Mrs. Grier waited in the office until two o'clock, when Dr. Moore arrived. He examined her and advised that she be placed under the influence of chloroform by means of an Eismark inhaler. One tooth was extracted by Dr. Sherraden, and, as is usual, the pain brought the patient to consciousness. Dr. Moore again placed the inhaler to her face and the dentist extracted another tooth. Instead of struggling as she did before Mrs. Grier lay perfectly quiet, and Dr. Moore, who was holding her hands, noticed that she stopped breathing. As this is not unusual in such cases, Dr. Moore was not alarmed, and told Dr. Sherraden to hasten the extraction of another tooth, thinking that the pain would bring the woman to consciousness. The tooth was extracted, but after giving two or three gasps the woman lay dead.

This is the statement made by Drs. Sherraden & Moore. "I have placed hundreds of patients under the influence of chloroform in thirty years' practice," said Dr. Moore last night, "and this is the first time I have had a fatal result. If I had treated the woman in the old way, and as many physicians do now, by placing a handkerchief saturated with the drug under her nose, or dropped the drug from a bottle, I might blame myself, but I did not do that, and have not done so for some time. I used the latest method approved by physicians, the Eismarck inhaler. Moreover I used a very small quantity of chloroform, less than a teaspoonful. I have attended the woman and never found that she was affected with heart disease, and I found her in a perfectly healthy condition when I examined her before the operation. I am anxious to have the case investigated, as I cannot see where I can in any way be blamed."

The inquest over the remains of Mrs. Grier will be held this morning at 11 o'clock. A post mortem examination may be held.—Omaha Daily, August 2d, 1895.

(The cause of this death—judging from the evidence herein contained—would seem to have been syncope with partial narcosis. Had the administrator on the discovery that the patient showed extreme palor and that the functions of life had in a measure ceased, quickly held the patient up by the heels and thrown cold water in her face, or had quickly applied the galvanic battery, she might be still living. But any delay in such cases is very likely to bring fatal results. By describing the effects of the anæsthetic to the patient before administering, ofttimes prevents the syncope as caused by fright. Ed.)—*Microcosm.*

